# Spatial–temporal risk of *Opisthorchis felineus* infection in Western Siberia and the Ural Region of Russian Federation: a joint Bayesian modelling study based on survey and surveillance data

**DOI:** 10.1186/s40249-025-01363-z

**Published:** 2025-09-22

**Authors:** Wen-Long Zhang, Yuan-Hong Zeng, Ying-Si Lai

**Affiliations:** 1 Department of Medical Statistics, School of Public Health, Sun Yat-Sen University, Guangzhou, Guangdong People’s Republic of China; 2 Sun Yat-Sen Global Health Institute, Sun Yat-Sen University, Guangzhou, Guangdong People’s Republic of China; 3 Health Information Research Center, Guangdong Key Laboratory of Medicine, School of Public Health, Sun Yat-Sen University, Guangzhou, Guangdong People’s Republic of China; 4 Guangzhou Joint Research Center for Disease Surveillance, Early Warning and Risk Assessment, Guangzhou, Guangdong People’s Republic of China

**Keywords:** *Opisthorchis felineus*, Western Siberia and the Ural Region, Bayesian geostatistical modeling, Joint analysis, Survey and surveillance data, High resolution risk mapping

## Abstract

**Background:**

Opisthorchiasis infected by *Opisthorchis felineus* has represented a significant but understudied public health issue for the population residing in Western Siberia and the Ural Region of the Russian Federation. This study aimed to produce high-resolution spatial–temporal disease risk maps for guiding prevention strategy in the above region.

**Methods:**

Data on prevalence and surveillance data reflecting reported annual incidence rate of *O. felineus* infection in the study region were collected through systematic review and the annual reports of the Ministry of Health of the Russian Federation. Environmental, socioeconomic and demographic data were downloaded from different open-access data sources. An advanced multivariate Bayesian geostatistical modeling approach was developed to estimate the *O. felineus* infection risk at high-resolution spatial–temporal by joint analysis of survey and surveillance data, incorporating potential influencing factors and spatial–temporal random effects. The annual spatial–temporal risk maps of *O. felineus* infection at a resolution of 5 × 5 km^2^ were produced.

**Results:**

The final dataset included 76 locations of survey data and 303 locations of surveillance data on *O. felineus* infection. The infection risk was high (> 25%) in most part of central and eastern regions, and relatively low (< 25%) in most part of western region, while temporal variations were observed across the sub-regions in recent decades. Particularly, in the densely populated eastern region, there was an increased trend of infection risk from 30.46% (95% Bayesian credible intervals, BCI 10.78–53.45%) in 1980 to 53.39% (95% BCI 13.77–91.93%) in 2019 and gradually transformed into high-risk. In the study region (excluding the western region due to data sparsity), the population-adjusted estimated prevalence was 46.61% (95% BCI 15.09–76.50%) in 2019, corresponding to approximately 7.91 million (95% BCI 2.56–12.98 million) people infected.

**Conclusions:**

The high-resolution risk maps of *O. felineus* in Western Siberia and the Ural Region of the Russian Federation have effectively captured the risk profiles, suggesting the infection risk remains high in recent years and providing substantial evidence for spatial-target control and preventive strategies.

**Supplementary Information:**

The online version contains supplementary material available at 10.1186/s40249-025-01363-z.

## Background

Opisthorchiasis, a food-borne trematodiasis, is a key focus for control in the World Health Organization (WHO)’s list of neglected tropical diseases initiative from 2021 to 2030 [1, 2]. Two species cause opisthorchiasis, namely *Opisthorchis viverrini*, which is endemic in Southeast Asia, and *O. felineus* (also known as the Siberian liver fluke or cat liver fluke), which is endemic in Eastern Europe [3, 4]. Particularly, Western Siberia and the Ural Region of Russian Federation, which consist of several administrative areas rather than an official composite region, are the world’s most important endemic regions for opisthorchiasis caused by *O. felineus* infection (Appendix B) [5–8]. The infection can lead to liver and biliary system-related diseases, such as cholelithiasis and cholangitis [9–11]. Recent research suggested that repetitive and persistent infection with *O. felineus* is a risk factor for the development of cholangiocarcinoma [11–14]. Besides, no effective control programs had been conducted by either the Russian Federation or the local Ministry of Health [15].

The slow-flowing rivers and expansive wetlands crossing the West Siberian Plain create a favorable environment for the life cycle of *O. felineus* within the first intermediate hosts (the freshwater snail of the genus Bithynia) and second intermediate hosts (cyprinoid fish). This has led to the high prevalence of commercial fish species infected with *O. felineus* metacercaria in the Ob-Irtysh Basin [16]. And local residents, the final host, get infection mainly through consumption of undercooked or raw freshwater fish infected with *O. felineus* metacercaria [9–11]. Potential environmental, socioeconomic and behavioral factors that influence the transmission of *O. felineus* or the distribution of the intermediate hosts, ultimately affect the endemicity of opisthorchiasis [4].

*O. felineus* infection has represented a significant but understudied public health issue for the population residing in Western Siberia and the Ural Region [17]. The raw prevalence of *O. felineus* infection was around 35% in Western Siberia from 1980 to 2000, suggesting high endemic in the region [18]. Another community survey conducted in 2016 reported varying and high prevalence of *O. felineus* infection, ranging from 50 to 90%, in 9 villages in the Tomsk Oblast (Western Siberia, Russia) [18, 19]. Additionally, the annual incidence rate of *O. felineus* across study region (Western Siberia and the Ural Region of Russian Federation) showing reasonable spatial consistency with prevalence rate, was reported to be 10 to 30 times higher than the national average level [18]. However, neither detailed spatial distribution nor temporal changes of *O. felineus* infection in the above region have been comprehensively studied yet.

Understanding the spatial–temporal distribution of *O. felineus* infection assists in disease control by guiding targeted interventions towards areas that most needed, optimizing efforts to reduce the disease burden. Bayesian geostatistical modeling is a highly rigorous framework widely used to produce accurate and reliable high-resolution risk maps for food-borne trematodiases [20, 21]. This approach models disease data with potential influencing factors and incorporates spatial–temporal random effects to estimate disease risk in areas without observations [22, 23]. Usually, disease data, mostly collected through cross-sectional studies within a specific population at a particular point in time, of the above model are based on point-referenced prevalence data, while the disease survey data are relatively sparse for the study region. On the other hand, the Ministry of Health of the Russian Federation officially released surveillance data, typically summarized at the first administrative division level (ADM1-level divisions). These data usually consist of numbers of incidents and case reports over a defined duration and province, providing valuable information for accurately predicting infection risk based on the spatial consistency of prevalence and annual incidence rate. However, the development of more advanced geostatistical models is necessary.

In this study, we aimed to develop an advanced spatial–temporal Bayesian geostatistical joint modeling approach to utilize both survey and surveillance data, to estimate spatial–temporal *O. felineus* infection risk in Western Siberia and the Ural Region of the Russian Federation at high spatial resolution, which further provides valuable information for spatial targeting of opisthorchiasis control interventions.

## Methods

### Ethics statement

This study incorporated survey data and surveillance data on opisthorchiasis, sourced from peer-reviewed published literature and annual reports from the regional departments of the Federal Service for Supervision of Consumer Rights Protection and Human Wellbeing (Rospotrebnadzor), respectively. The original sources included statements indicating that appropriate ethics approvals were obtained. The data used in this study were aggregated at either village/community or areal level and did not include any personally identifiable information at individual or household levels. Therefore, no specific ethical concerns were identified in this research.

### Disease data

We conducted a systematic review to collect relevant prevalence data of opisthorchiasis in Western Siberia and the Ural Region of Russian Federation from inception to August 31, 2024 (registered in the International Prospective Register of Systematic Reviews NO. CRD42023445574), and reported it following the Preferred Reporting Items for Systematic Reviews and Meta-Analyses‌ (PRISMA) guidelines (Appendix A). Two general databases (i.e., ISI Web of Science and PubMed) and two local databases (i.e., eLIBRARY and Scientific Medical Library of the Siberian State Medical University) were searched with search terms “(liver fluke* OR Opisthorchi*) AND (Russia* OR RF OR Siberia OR Ural)” (Appendix C). For literature in Russian language, we enlisted the assistance of a student proficient in Russian for translation purposes, while also utilizing translation software to ensure accuracy and comprehensiveness. In our search strategy, we did not impose any restrictions on language, survey date, or study design. Protocol for the inclusion, exclusion, and extraction of survey data is provided in Appendix C, referenced to Zhao et al.’s protocol on systematic review of opisthorchiasis in Southeast Asia [20]. Briefly, we included community-based surveys that provided prevalence related data (i.e., reporting number of examined and number of positive, or prevalence). All the included records, with survey locations georeferenced by Google Maps, were extracted into a database with detailed information (e.g., literature information, survey information, location information, and disease-related information), and were verified by two independent reviewers subsequently. Finally, the quality assessment of each included literature was undertaken using a nine-point checklist (Appendix D).

Surveillance data were obtained from the annual reports of the Ministry of Health of the Russian Federation. It was accessible from the regional departments’ webpages of the Federal Service for Supervision in Consumer Rights Protection and Welfare (Appendix E). Data were included if it contained at least one of the followings: the annual number of positive cases reported or the annual incidence rate. Data lacking both of that information was excluded. Details protocols are provided in Appendix C.

### Environmental, socioeconomic, and demographic data

Environmental data (i.e., land surface temperature (LST) in the daytime, normalized difference vegetation index (NDVI), land cover, elevation, annual precipitation, distance to the nearest open water bodies and soil moisture), socioeconomic data (i.e., human influence index (HII), travel time to the nearest big city), and demographic data (i.e., population count, population growth rate) were obtained from the open-access databases (Appendix F) [24]. They were further integrated into a regular grid with a spatial resolution of 5 $\times$ 5 km^2^. As the collected high-resolution data on potential influencing factors did not cover the entire study period (1980 − 2019), the average value of each factor within each grid pixel over available years was utilized. In addition, similar classes of land cover were re-grouped into five categories, that is (1) croplands; (2) forests; (3) shrub and grass; (4) urban; and (5) others. As high-spatial resolution demographic data before the year 2000 were lacking, the population counts were estimated based on the nearest available year of population data and the population growth rate. The formula used for estimation was ${P}_{m}= {P}_{n}* {e}^{\left(m-n\right)*{rate}_{n}}$. Here ${P}_{m}$ and ${P}_{n}$ are the population counts in year $m$ and $n$ ($m$ > $n$), respectively.

### Statistical analysis

#### Data preprocessing and variable selection

Multi-categorical predictors were converted into dummy variables, and continuous predictors were standardized with a mean of zero and a standard deviation of one. To prevent collinearity, we calculated Pearson’s or Spearman correlation coefficients for each pair of continuous variables. If the absolute values of correlation coefficients exceeded 0.8, we only retained the one more meaningful or with higher-quality in each pair [25].

To construct a parsimonious model, Bayesian variable selection was utilized to determine the optimal set of predictors. First, in order to identify the optimal functional form (continuous or categorical) for the continuous predictors, they were converted into two-level or three-level categorical variables based on preliminary, exploratory, and graphical analysis [26]. Bayesian geostatistical models, introduced in the following part, were constructed using either form as the independent variable. The functional form with the minimum Deviance Information Criterion (DIC) was chosen as the best option. Second, all possible combination subsets of variables were considered to identify the best combinations of predictors. And the one with the lowest DIC was selected in the final model. Particularly, the distance to the nearest open water bodies was included in all predictor combinations, based on the previous studies that demonstrated its importance on liver fluke infection [27]. The variable selection process was conducted within the framework of the spatial–temporal model for survey data.

#### Model fitting, validation and risk mapping

An advanced multivariate Bayesian geostatistical joint modelling approach was developed [28, 29], to jointly analyze both survey and surveillance data, as well as data at both point- and areal levels, that is,

1 $$\left\{\begin{array}{c}logit\left({p}_{it}\right)={\beta }_{0}+{{\varvec{x}}}_{{\varvec{i}}{\varvec{t}}}^{\boldsymbol{^{\prime}}}\beta +\omega \left(s, t\right)+{\varphi }_{i}, i=1,\dots ,{n}_{p}\#\left(a\right)\\ logit\left({p}_{it}\right)={\beta }_{0}+\widetilde{{{\varvec{x}}}_{{\varvec{i}}{\varvec{t}}}^{\boldsymbol{^{\prime}}}}\beta +{\left|{A}_{i}\right|}^{-1}{\int }_{Ai}\omega \left(s, t\right)dxdt+{\varphi }_{i}, i={n}_{P+1},\dots ,{n}_{P}+{n}_{A}\#\left(b\right)\\ log\left({\upgamma }_{jt}\right)={\alpha }_{0}+\alpha {\left|{A}_{j}\right|}^{-1}{\int }_{Aj}\omega \left(s,t\right)dxdt+{\phi }_{j} j=1,\dots ,17\#\left(c\right)\end{array}\right.$$

For survey data, we assumed the number of positive ${Y}_{it}$ following a binomial distribution ${Y}_{it} \sim Bin\left({p}_{it}, {n}_{it}\right)$, where ${p}_{it}$ and ${n}_{it}$ denoted the probability of infection and the number of examined at location $i$ in survey year $t$, respectively. $i$ represented either the location of point-referenced survey data or the area for area-level survey data. Allow $i={1,\dots ,n}_{P},{n}_{P+1},\dots ,{n}_{P}+{n}_{A}$, where ${n}_{P}$ was the total number of locations for point-referenced surveys and ${n}_{A}$ was the total number of areas for areal level surveys, respectively. ${p}_{it}$ was modeled on the logit scale.

In terms of point-referenced prevalence data [Formula 1, (a)], where$i={1,\dots ,n}_{P}$, and ${{\varvec{x}}}_{{\varvec{i}}{\varvec{t}}}^{\boldsymbol{^{\prime}}}$ represented the vector of covariates, and ${\beta }_{0}$ and ${\varvec{\beta}}$ were the intercept and corresponding vector of regression coefficients, respectively. Under the presumption of spatial–temporal dependence among survey outcomes, we defined the $\omega \left(s, t\right)$ as a spatial–temporal random effect for the ${i}^{th}$ location in survey year$t$. The spatial–temporal random effect was assumed to follow a zero-mean Gaussian distribution, depicting the spatial correlation between locations by a stationary Matérn covariance function and temporal correlation between different survey years through autoregressive order 1 (AR1), respectively. To reduce computational burden, regular temporal knots were set every 11 years, that is,$\omega \left({\omega }_{t}=1975, {\omega }_{t}=1986{, \omega }_{t}=1997,{\omega }_{t}=2008,{\omega }_{t}=2019\right)$. The latent fields for remaining years were approximated by projecting $\omega$ using a B-spline basis function of degree two [30, 31]. ${\varphi }_{i}$ was the exchangeable non-spatial random effect following a zero-mean normal distribution${\varphi }_{i} \sim N\left(0, {\sigma }_{nonsp}^{2}\right)$. As for area-level prevalence data (Formula 1, (b)), where $i={n}_{P+1},\dots ,{n}_{P}+{n}_{A}$, $\widetilde{{{\varvec{x}}}_{{\varvec{i}}{\varvec{t}}}^{\boldsymbol{^{\prime}}}}$, $\widetilde{{{\varvec{x}}}_{{\varvec{i}}{\varvec{t}}}^{\boldsymbol{^{\prime}}}}$ and ${\left|{A}_{i}\right|}^{-1}{\int }_{Ai}x\left(s,t\right)dxdt$ were the vector of average values of covariates and the average spatial–temporal random effects of pixels within the ${i}^{th}$ area in survey year$t$. $\left|{A}_{i}\right|={\int }_{{A}_{i}}1ds$ indicates the size of ${i}^{th}$ area.

For area-level surveillance data [Formula 1, (c)], we assumed the reported number of cases ${\gamma }_{jt}$ followed a Poisson distribution, ${\gamma }_{jt} \sim Poissin\left({\lambda }_{jt}\right)$, where ${\lambda }_{jt}$ denoted the expected number of cases in ${j}^{th}$ area in reported year $t$. In cases of insufficient survey data, surveillance data can additionally provide information on spatial characteristics based on the similarity of spatial patterns of the two kinds of data. As we assumed spatial consistency between survey and surveillance data, share component of spatial–temporal random field $\omega \left(s,t\right)$ was introduced. And $\alpha$ named the tuning coefficient, serving as a weight on the shared component to account for the differences in scale between the two sub-models. ${\alpha }_{0}$ was the intercept. The joint model was developed under a Bayesian framework and Integrated Nested Laplace Approximation-Stochastic Partial Differential Equation (INLA-SPDE) approach was used for model fitting, and we calculated Bayesian credible intervals (BCI) to reflect the uncertainty of the results. Additionally, sensitivity analysis was conducted to assess the robustness of the joint model (Appendix L).

Leave-one-out cross-validation (LOOCV) approach was conducted for model validation. We calculated the area under the receiver-operating characteristic (ROC) curve (AUC) to assess the model performance [21, 32, 33]. A regular grid with a spatial resolution of 5 × 5 km^2^ was overlaid across Western Siberia and the Ural Region, resulting in 305,463 pixels [34]. The infection risk for each pixel was estimated annually from 1975 to 2019 using Bayesian kriging. Low, moderate, and high infection risk areas were defined by prevalence rates of $<$ 5%, 5–20%, and $>$ 20%, respectively, based on the WHO’s recommendation [35]. All the statistical process was done in R 4.3.1 (The R Foundation, Auckland, New Zealand) and risk maps were produced using ArcGIS 10.8.1 (Esri, Redlands, The United States). The provincial and regional infection risk were calculated based on population-weighted pixel-level infection risk.

## Results

### Summary of observed data

**Fig. 1:**
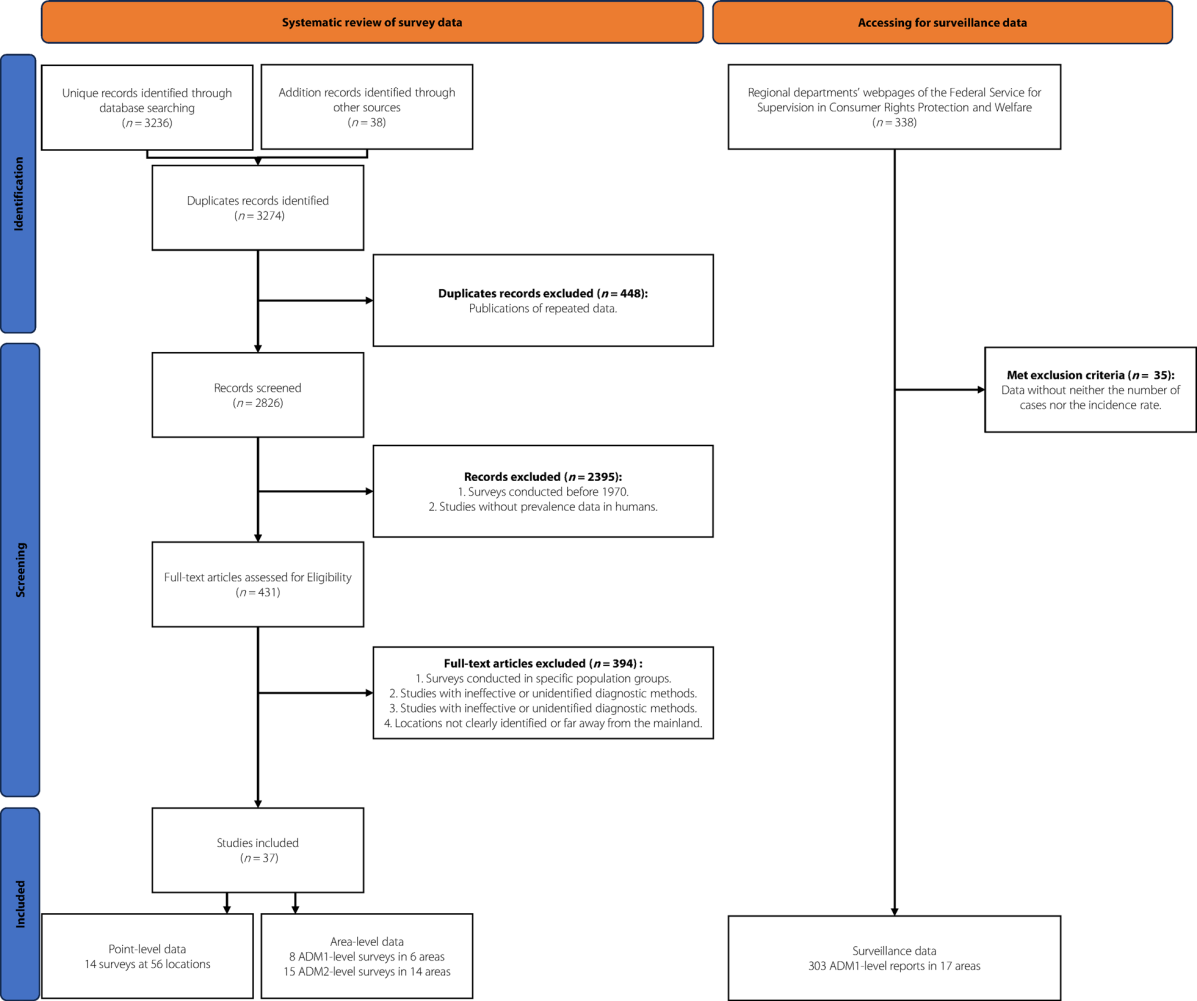
Data selection and collection flow chart

**Fig. 2:**
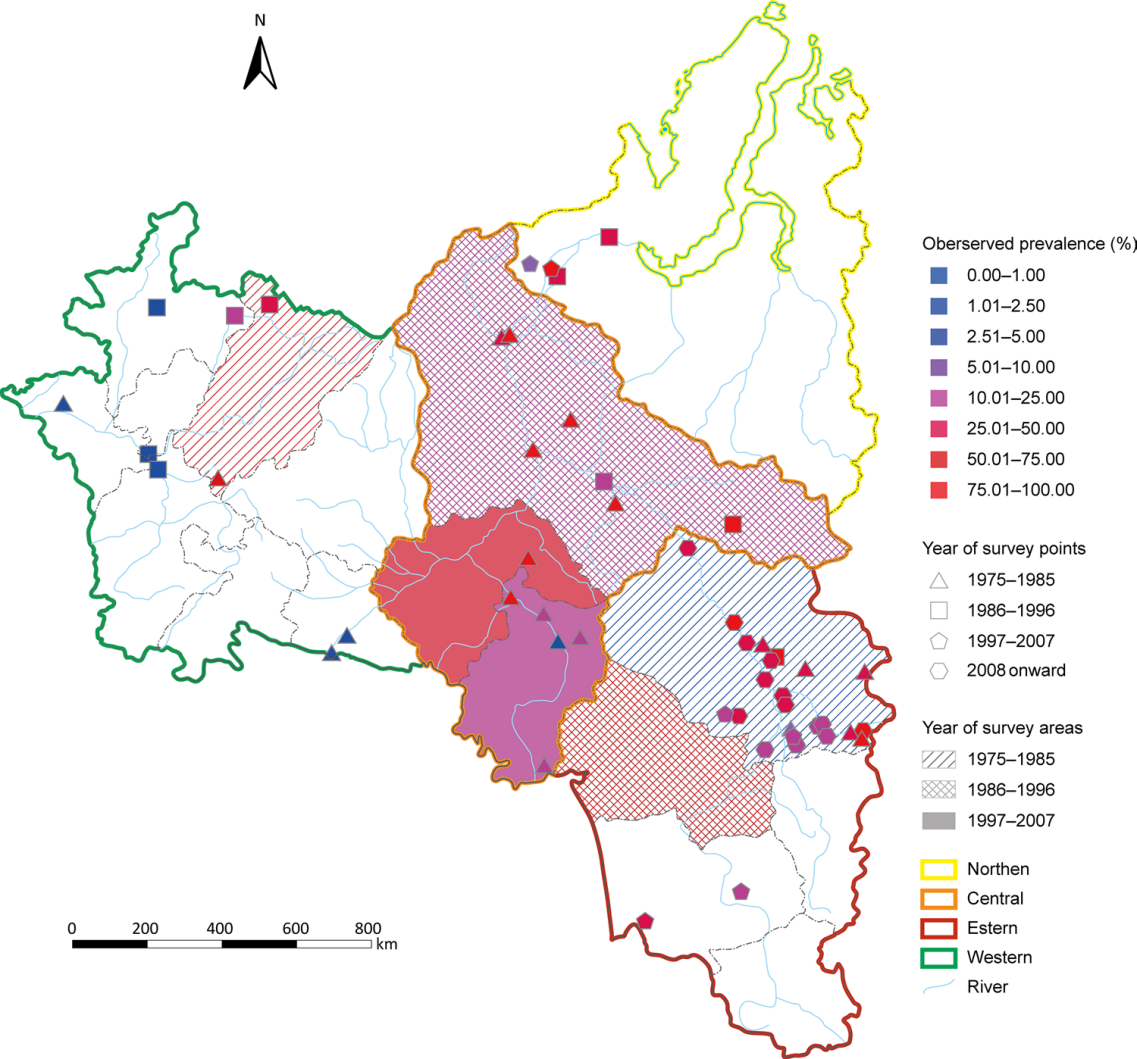
Survey locations and observed prevalence of *Opisthorchis felineus* infection in Western Siberia and the Ural Region of Russian Federation. Map approval No.: GS (2025) 3546

Through systematic review of peer-reviewed literature, we identified 3170 references. And 38 additional references were gathered from other sources. In accordance with the predefined inclusion and exclusion criteria, a comprehensive analysis yielded a total of 78 eligible records, resulting in 8 surveys conducted across 6 administrative divisions of level one (ADM1-level divisions), 15 surveys from 14 ADM2-level divisions, 14 surveys at 56 point-referenced locations, and 303 surveillance data at 17 ADM1-level divisions, as shown in Table 1. A flow chart for data collection process was presented in Fig. 1. The geographic locations of the observed prevalence and the annual incidence rate of *O. felineus* infection were shown in Fig. 2 and Appendix H, respectively. Approximately 87% of the surveys were conducted in the central and eastern of Western Siberia and the Ural Region, with a notable focus on Tomsk Oblast (48%) and Khanty-Mansi Autonomous Okrug (14%). Survey data were lacking for 2 geographical units in the southeastern part, namely the Republic of Altai and Kemerovo Oblast, and 3 in the western part, namely the Republic of Udmurtia, Sverdlovsk Oblast, and Chelyabinsk Oblast. All surveys were conducted after 1975, nearly 55% were done before 2000. Around 79% of surveys employed the stool microscopy technique for diagnosis. The highest prevalence observed was 90%, recorded in Tomsk Oblast (2016), while the lowest was 0.4%, reported in the Republic of Tatarstan (1985). The median prevalence (upper and lower quartiles) was 31.60% (19.23%, 51.42%). Additionally, the median (upper and lower quartiles) of the reported annual incidence rate was 64.45 (6.64, 135.70) per 100,000. Nearly 50% of reported cases were concentrated in the Khanty-Mansi and Yamalo-Nenets Autonomous Okrug, while only 6% were reported in the 8 geographical units of the Western Ural Region. The spatial risk pattern of surveillance data showed consistency with that of the survey data. Maps of observed data are provided in Fig. 2.

**Table 1:** Overview of *Opisthorchis felineus* infection survey data in western Siberia and the Ural Region of Russian Federation

Year of survey	1975–1985	1986–1996	1997–2007	2008 onward	Total
Number of articles	16	9	8	6	39
Number of surveys/locations^a^	28/27	13/13	13/11	24/24	78/75
Location type					
ADM^b^1-level	3/3	2/2	3/2	0/0	8/7
ADM2-level	8/8	2/2	4/3	0/0	14/13
Point-level	17/16	9/9	6/6	24/24	56/55
Diagnostic methods					
Stool microscopy	26/25	13/13	9/7	14/14	62/59
Others^c^	1/1	0/0	4/4	9/9	14/14
Ns^d^	1/1	0/0	0/0	1/1	2/2
Raw prevalence (%)	4.53	36.94	21.27	36.87	6.74

^a^Presented as surveys/locations

^b^ADM: Administrative

^c^Others:Bile microscopy and unspecified

^d^Ns: not stated or missing

### Geostatistical modeling and validation

Four covariates were included in the final geostatistical model, as shown in Table 2. Negative associations were found for the infection risk with travel time to the nearest big city. Increase in 1 min in travel time to the nearest big city was associated with the 0.53 (95% BCI 0.04–1.02) decrease in the logit of the prevalence. There is a 95.21% confidence to believe that people living in areas with a high level of NDVI (> 0.41) had a higher infection risk (Odds Ratio, *OR*: 1.69, 95% BCI 0.92–3.13), compared to those in areas with NDVI ≤ 0.41. Both the distance to the nearest open water bodies and elevation were found to be statistically insignificant, based on the two-sided 95% BCIs including zero. Model validation showed that the AUC of the ROC was 0.71, suggesting a reasonable capacity for estimation accuracy.

### Spatial–temporal risk profiles

**Fig. 3:**
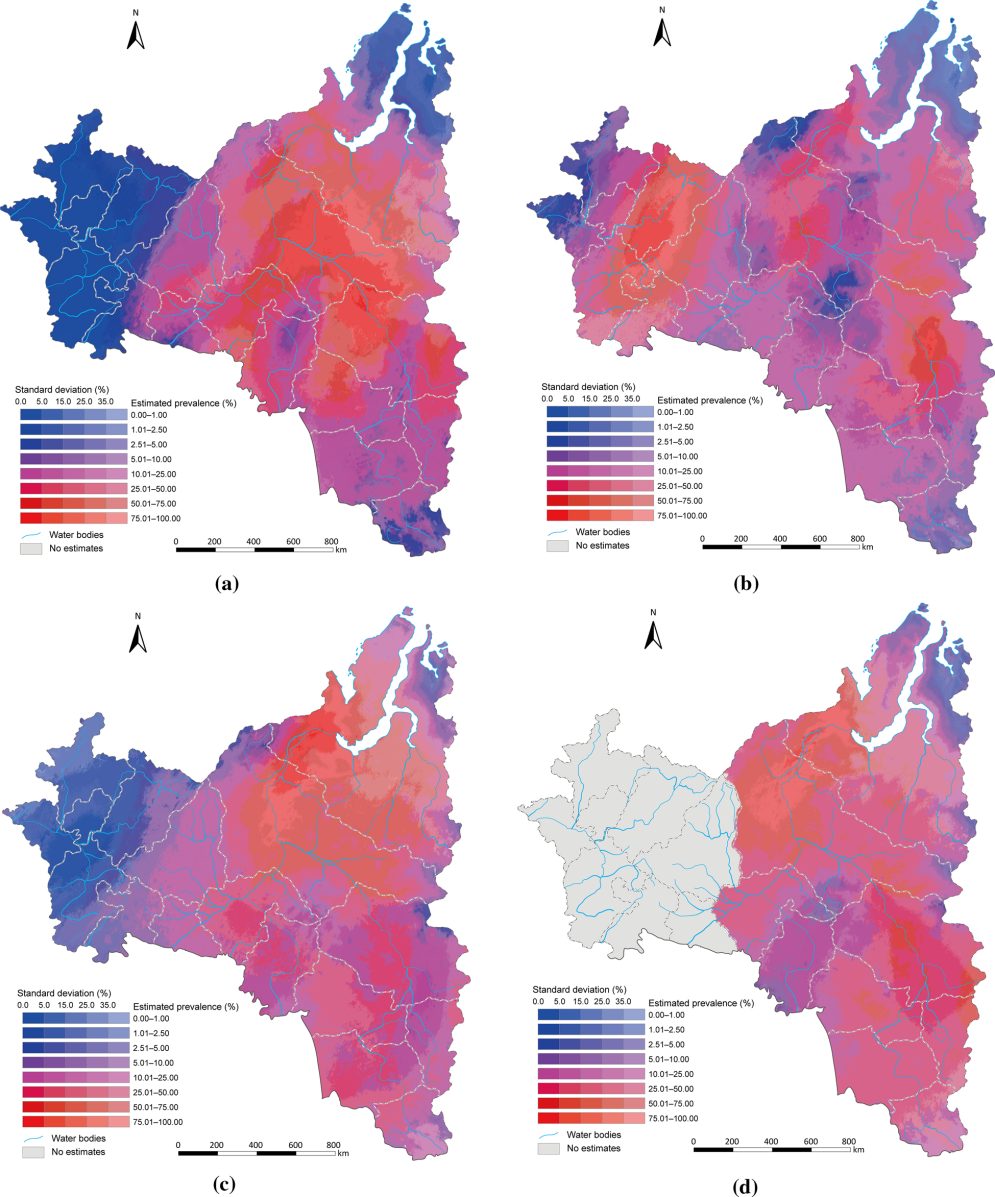

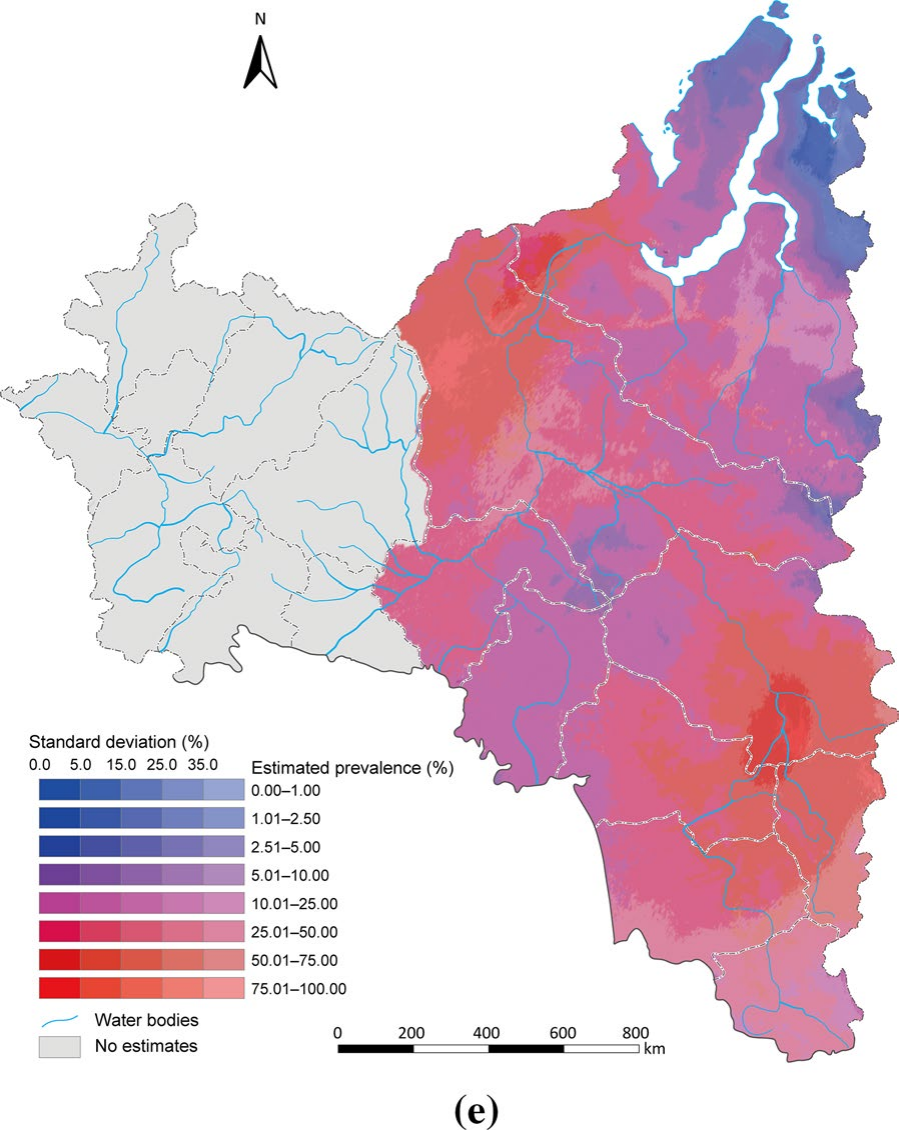
Model-based estimated risk maps depict the prevalence of *Opisthorchis felineus* infection across Western Siberia and the Ural Region of the Russian Federation. Estimation was excluded for western region after 2010 due to data sparsity. Estimated prevalence based on the median and standard deviation of the posterior estimated distribution of infection risk in (**a**) 1980, (**b**) 1990, (**c**) 2000, (**d**) 2010, (**e**) 2019. Map approval No.: GS (2025) 3546

Estimated risk maps of *O. felineus* infection in different year were presented in Fig. 3. Overall, infection risk was high (> 25%) in most part of central and eastern regions, low (< 25%) in most part of western region, while the temporal variances in infection risk were shown across different areas. In the central region, there was a decreased trend of infection risk from 46.94% (95% BCI 22.97–69.97%) to 26.27% (95% BCI 6.93–68.81%) until 1990, an increased trend to 41.31% (95% BCI 16.71–70.15%) until 2000, and then a decreased trend to approximately 35% in 2019. There was a decreasing trend in infection risk in the central part (e.g., the basin of the Ob River within the Khanty-Mansi Autonomous Okrug) until 2000, followed by an increasing trend up to 2010, and a gradual decreasing trend since then. The infection risk of northern region remained approximately 30% from 1980 to 1990, and there was an increased trend to approximately 75% until 2000, and then a decreased trend to approximately 30% in 2019. The northern part (e.g., Yamalo-Nenets Autonomous Okrug) of the Arctic Circle section remained a low-risk infection area throughout the entire study period, while the densely populated areas (e.g., Shuryshkarsky District) exhibited similarly to the central region. In the western part (e.g., Republic of Udmurtia, Republic of Tatarstan, Republic of Bashkortostan, Perm Krai, Kirov Oblast), there was an increasing trend until 1990, followed by a decreasing trend until 2000. Projections for subsequent years in the Ural region have not been conducted due to insufficient data. Particularly, in densely populated eastern region (e.g., the vicinity of the city of Tomsk in Tomsk Oblast), there was an increased trend of infection risk from 30.46% (95% BCI 10.78–53.45%) to 53.39% (95% BCI 13.77–91.93%) in 2019 and gradually transformed into high-risk. The estimation uncertainties (shown as standard deviation of posterior risk distribution) remained high (> 35%) in most areas of northern regions, while relatively low in central and eastern regions (Fig. 3).

### Estimates of number of people infected

**Fig. 4:**
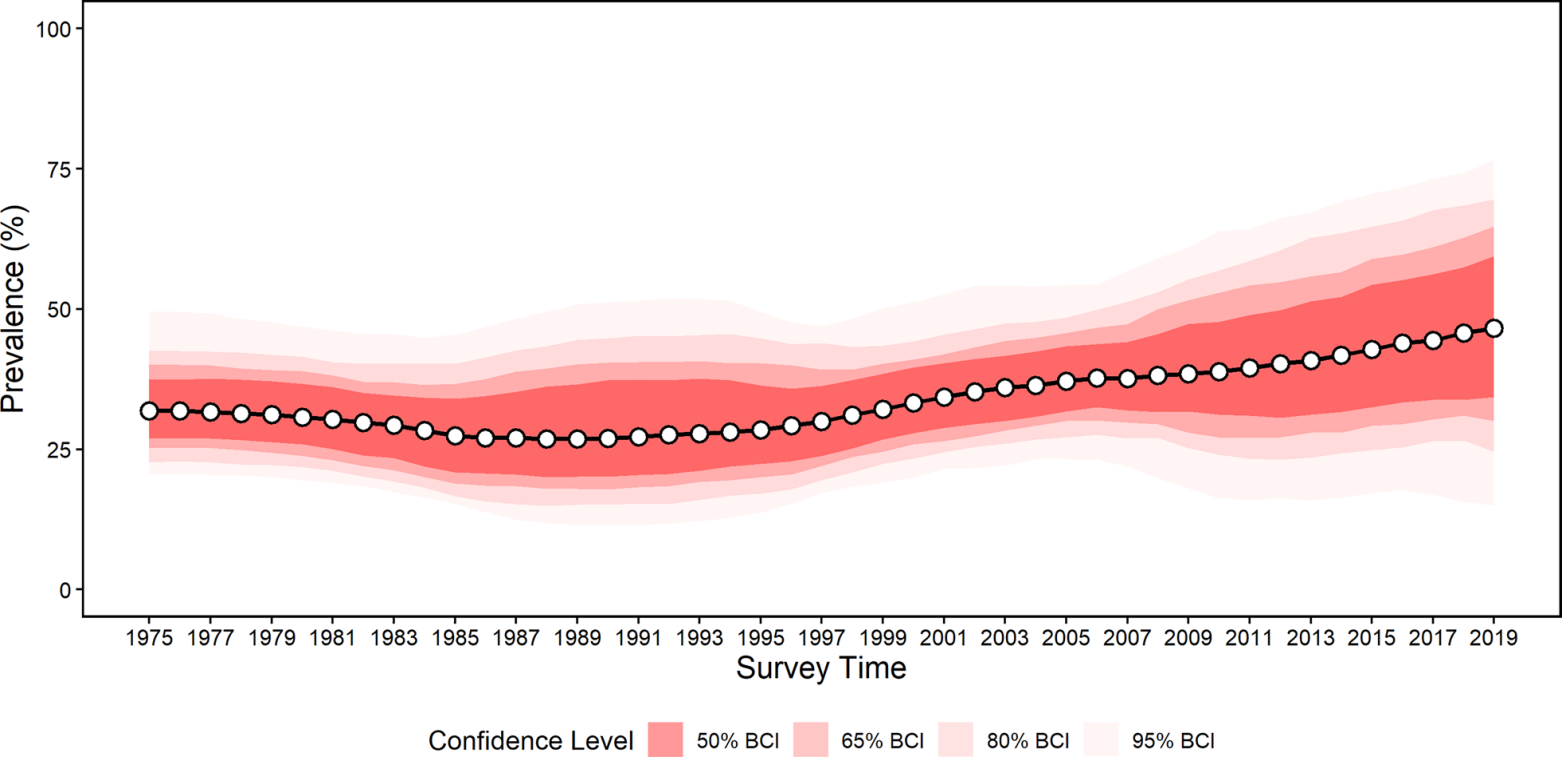
Trends in the estimated prevalence of *Opisthorchis felineus* infection in Western Siberia and the Ural Region (excluding the western region due to data sparsity). BCI: Bayesian credible interval

Due to insufficient data, estimates of total infection risk in the study region did not include the western part. Overall, the regional population-adjusted estimated prevalence remained a consistently high level. It showed a slightly decreasing trend from 31.86% (95% BCI 20.40–49.60%) in 1975 to 26.82% (95% BCI 11.86–49.52%) in 1988, followed by an upward trend ever since (Fig. 4). Additionally, the population-adjusted prevalence was estimated at 46.61% (95% BCI 15.09–76.5%), and around 7.91 (95% BCI 2.56–12.98) million people in Western Siberia and the Ural Region (excluding the western region) were infected with *O. felineus* in 2019. In particular, the highest prevalence rate was estimated in Tomsk Oblast, reaching 65.51% (95% BCI 20.22–91.72%), with 0.77 (95% BCI 0.22–0.98) million people infected in 2019.

## Discussion

To our knowledge, we presented the first high-resolution (5 × 5 km^2^) model-based risk maps for *O. felineus*-induced opisthorchiasis in Western Siberia and the Ural Region of the Russian Federation, using an advanced multivariate Bayesian geostatistical joint modeling approach to integrate both survey and surveillance data, as well as disease-related covariates. Our findings will be important for guiding the spatially targeted control measures to reduce the burden of this disease.

Combining the estimated high-resolution spatial–temporal maps with overall population-adjusted prevalence trends suggested that the risk of *O. felineus* infection remained high and continued to show an expanding trend. The temporal trend of prevalence in different sub-regions indicated, the temporal trends were consistent with the results reported by Armignacco and Pozio, indicating a westward spread from Western Siberia (Appendix K) [36, 37]. Official data also corroborated this trend [38]. In the years 1980 to 2000, our predictions and the observed ADM1-level maps produced by Fedorova et al. exhibit similar spatial patterns [18]. After 2000, the main areas of moderate-to-high infection risk primarily concentrated along the Ob River basin. The prolonged high prevalence level was probably attributed to local factors, particularly the dietary habits of residents in the Ob River basin consuming raw fish and thriving livestock industry, as well as the absence of systematic and continuous disease control programs implemented by either the Russian Federation or the local Ministry of Health [27, 39]. Moreover, the infection rate of edible commercial fish in the Ob River with *O. felineus* is also extremely high. For instance, cyprinid fish, such as carp, were reported to have an infection rate of up to 100%, which provides a suitable environment for the spread of *O. felineus* [16]. These findings suggest that the focus of prevention and control efforts should be placed on the upper and lower basins of the Ob River. Given increased trade and travel in Western Siberia, *O. felineus* infection may potentially spread to adjacent endemic regions [38]. In the northern region of the Yamalo-Nenets Autonomous Okrug, which is located deep within the Arctic Circle and is sparsely populated, the infection risk was low. It may be attributed to the presence of permafrost and ice-covered rivers throughout the year, so that the local residents preferred consuming marine fish over freshwater fish. To be noted, different time trends have been observed between the population-adjusted estimated prevalence and annual incidence rate. Indeed, even both prevalence and surveillance data showed spatial consistency, the latter based on reported cases, influenced by frequent annual fluctuations and data collection biases, may not accurately reflect the temporal risk. However, incidence data can reflect some spatiotemporal aspects of disease information by joint model. Therefore, the joint model can leverage the overall strengths of both data types to compensate the limitations of sparse data. Moreover, the joint model only borrows a portion, rather than all, of the incidence information, which can be reflected by the spatial–temporal random effect [28, 29]. The results of sensitivity analysis indicated that the joint model outperformed the prevalence-based model, in terms of mean absolute error (MAE), mean square error (MSE), and AUC (Appendix L). Therefore, we believed the joint model shared information in data-sparse settings and provided a more comprehensive risk assessment.

We collected several socioeconomic and environmental predictors, and selected the appropriate combinations of potential predictors by the backward elimination approach, which may provide insights in prevention and control of the *O. felineus* infection. We identified a negative association between travel time to the nearest big city and the infection risk, perhaps due to individuals residing in areas closer to urban areas, where there is a longer history and culture of consuming raw fish deeply ingrained. Local residents of Melnikovo, the administrative center of the Shegarsky District in Tomsk Oblast that perceive *O. felineus* infection as a common trait among the population, which could partially explain our findings [27]. Additionally, considering the population of Western Siberia in Russia is predominantly concentrated in urban areas, there may be a bias in the selection of survey locations, which could potentially introduce bias into the study. Moreover, in areas with shorter travel times and higher economic development, local residents are more likely to buy expensive but raw fish, which may further influence infection risks. In addition, it is highly likely that the categorical form of NDVI exhibits a positive trend with infection risk, which is consistent with a previous study conducted in South Korea of *Clonorchis sinensis*, another major species of liver flukes [21]. This suggests that areas with higher vegetation cover may also have a higher risk of *O. felineus*, which is possibly because regions with dense vegetation are typically associated with more developed livestock industries, implying a greater number of reservoir hosts (e.g. cats, cattle, and sheep), which facilitates the spread of *O. felineus*. Additionally, our data suggest, areas with high vegetation cover are often marshy regions, these slow-moving water bodies provide favorable living conditions for the development of *O. felineus* eggs into mature cercariae. These cercariae can easily enter freshwater fish, especially in the gentle marsh areas, which are then captured and consumed by humans. Moreover, previous research has indicated that in certain sections of the rivers within the study region, the infection rate of some fish species with *O. felineus* can reach up to 100% [16]. However, infection risk and distance to the nearest open water bodies showed no significant association in this study, which may be attributed to the fact that most observed data were distributed in areas with abundant rivers and lakes. Actually, 98.13% (299,751/305,463) of the pixels were located within 0.5 km of the nearest water body. Elevation also appears to be insignificant concerning infection risk, possibly because the majority of the population (around 90%) resided in low-altitude plain areas below 300 m.

A significant innovation of this study lies in the high-resolution spatial risk estimation for liver fluke, where, similar spatial effects were shared between annual incidence rate and prevalence data. The study region is characterized by vast territories and sparse populations, and despite the severity of the parasite, it is often overlooked and underestimated, resulting in limited survey data collection. However, the Ministry of Health of the Russian Federation provides long-term annual incidence rate data reports. Therefore, a joint modeling approach was developed to integrate these two types of data, not only to analyze the spatial effects of the annual incidence rate but also to share them with the prevalence rate data. This would offer new perspectives to other diseases facing similar challenges in data collection. We also jointly analyzed survey data at point- and area-level to make use of all available data [30].

Indeed, this study has several limitations. Two variables potentially affecting *O. felineus* infection were identified. However, the essential variable of raw fish consumption was not included due to data unavailable, leading to considerable uncertainty in our predictions. Obtaining this data in the future could improve estimation accuracy. For some years, especially before 2000, where corresponding influencing factors were lacking, we used the average of available data as a substitute. This decision was made considering that *O. felineus* infection is chronic and influenced by environmental and economic factors over a long period of time. The survey data collected for the study region is relatively sparse, despite our efforts to gather as much data as possible, resulting in a wide 95% BCI of prevalence. The estimated prevalence in sub-region over time revealed that the northern region exhibited extremely wide BCI due to sparse data (Appendix K). However, the overall trend of high-risk regions remained evident. Therefore, we contend that these results possess a degree of reference value and offer valuable insights for public health decision-making. Future research should focus more intently on high-risk areas, and strive for a more comprehensive collection of samples from additional sites especially in northern regions. As previously mentioned, our estimated prevalence 46.61% in Western Siberia and the Ural Region excluding the western region due to data sparsity is higher than the 35% reported in prior studies by meta-analysis, and the number of infected people (7.91 million) is higher than that reported (1.6 million) by WHO [35, 40, 41]. However, it should be noted that the data previous studies used were relatively dated. Since we incorporated the relevant data from previous studies and improved the method, the results may be reasonable. Besides, by adding incidence rate data to the model, we achieved a prediction accuracy of 0.71 AUC under the ROC curve, indicating the reliability of our results. We didn’t consider factors such as age and gender, as most survey data did not include clear age and gender groups. In addition, almost 80% of the diagnostic method employed in the included survey is stool microscopy (Table 2), but the specific stool examination technique was unspecified. Since liver fluke is typically diagnosed using Kato-Katz and formalin-ether concentration technique, the results might be inaccurate without specifying the stool examination method [42, 43].

**Table 2:** Posterior summaries of model parameters for *Opisthorchis felineus* infection

Variable	Estimated median (95% BCI^a^)	*OR*^b^ (95% BCI)	Prob^c^ (%)
Travel time to the nearest big city (min)	– 0.53(– 1.02, – 0.04)*	0.59 (0.36, 0.96)	2.80
Normalized difference vegetation index			
≤ 0.41	Ref.	Ref.	
> 0.41	0.53 (– 0.09, 1.14)	1.69 (0.92, 3.13)	95.21
Distance to the nearest open water bodies (km)			
≤ 0.018	Ref.	Ref.	
> 0.018	0.18 (– 0.56, 0.93)	1.20 (0.57, 2.52)	67.60
Elevation (m)			
≤ 89	Ref.	Ref.	
> 89	0.14 (– 0.57, 0.85)	1.15 (0.56, 2.34)	65.00
Range (km)	1251 (1151, 1369)	–	–
Spatial variance (${\sigma }_{\phi }^{2}$)	19.28 (15.78, 25.62)	–	–
Non-spatial variance for survey (${\sigma }_{nonsp1}^{2}$)	0.64 (0.59, 0.71)	–	–
Temporal correlation coefficient ($\rho$)	– 0.21 (– 0.26, 0.17)	–	–
Tuning coefficient ($\alpha$)	0.73 (0.64, 0.80)	–	–

^a^BCI Bayesian credible interval

^b^*OR*: Odds Ratio

^c^Posterior probability of *OR* > 1

^*^Significant correlation inferred from 95% Bayesian credible interval (not included zero)

^-^Model parameters, no need to estimate *OR*

## Conclusions

Our high-resolution risk maps of *O. felineus* in Western Siberia and the Ural Region of the Russian Federation have effectively captured the risk profiles. These maps reveal that the infection risk remains high in the study region in recent years. These findings provide substantial evidence for spatial-target control and preventive strategies to mitigate the impact of *O. felineus* infections in these regions.

## Supplementary Information

Supplementary Material 1.

## Data Availability

The source of disease data, socioeconomic, environmental, and demographic data were listed on Appendix C, Appendix E and Appendix F, respectively. All other data are available from the open-access databases.

## Abbreviations

*O. felineus*: *Opisthorchis felineus*
BCI: Bayesian credible intervals
WHO: World Health Organization
ADM: Administrative
PRISMA: Preferred reporting items for systematic reviews and meta-analyses‌
LST: Land surface temperature
NDVI: Normalized difference vegetation index
HII: Human influence index
DIC: Deviance information criterion
AR1: Autoregressive order 1
INLA-SPDE: Integrated nested Laplace approximation-stochastic partial differential equation
LOOCV: Leave-one-out cross-validation
ROC: Receiver-operating characteristic
AUC: Area under curve
*OR*: Odds ratio
MAE: Mean absolute error
MSE: Mean square error
