# Supplementary material for: Spatial–temporal risk of Opisthorchis felineus infection in Western Siberia and the Ural Region of Russian Federation: a joint Bayesian modelling study based on survey and surveillance data
Source: Infect Dis Poverty. 2025 Sep 22;14:95. doi: 10.1186/s40249-025-01363-z (PMC12452019; doi:10.1186/s40249-025-01363-z)
Supplement: Supplementary file 1 — Supplementary Material 1. [file 40249_2025_1363_MOESM1_ESM.docx]

## Supplementary appendix

**Supplement to: The Spatial-Temporal Risk of *Opisthorchis felineus* in Western Siberia and the Ural Region** **of Russian Federation: A Joint Bayesian Modelling Study Based on Survey and Surveillance Data**

**Contents**

[Appendix A: PRISMA 2020 4](#_Toc201567450)

[Supplementary File 1: PRISMA 2020 for Checklist 4](#_Toc201567451)

[Appendix B: Western Siberia and the Ural Region 7](#_Toc201567452)

[Supplementary File 2: Presentation of study area 7](#_Toc201567453)

[Supplementary Table 1: Administrative areas included in Western Siberia and the Ural Region. 7](#_Toc201567454)

[Appendix C: Study protocol 8](#_Toc201567455)

[Supplementary File 3: Study protocol 8](#_Toc201567456)

[Appendix D: Quality assessment of eligible studies 12](#_Toc201567457)

[Supplementary File 4: Quality assessment of eligible studies using an adapted nine-point quality assessment checklist 12](#_Toc201567458)

[Supplementary Figure 1: The quality assessment of eligible studies. 12](#_Toc201567459)

[Supplementary Table 2: Result of quality assessment for eligible literatures. 13](#_Toc201567460)

[Appendix E: Surveillance data sources 14](#_Toc201567461)

[Supplementary Table 3: Sources and access dates of surveillance data. 14](#_Toc201567462)

[Appendix F: Covariate 15](#_Toc201567463)

[Supplementary Table 4: Remote sensing data sources^a^ 15](#_Toc201567464)

[Supplementary Table 5: The spatial covariates used in the present study 16](#_Toc201567465)

[Appendix G: Model fitting 17](#_Toc201567466)

[Supplementary File 5: Model fitting 17](#_Toc201567467)

[Appendix H: Surveillance data 18](#_Toc201567468)

[Supplementary Table 6: Overview of *O. felineus* infection surveillance data in Western Siberia and the Ural Region of Russian Federation. 18](#_Toc201567469)

[Appendix I: The variable selection 19](#_Toc201567470)

[Supplementary File 6: The collinearity, optimal functional form and selection of variable. 19](#_Toc201567471)

[Supplementary Table 7: The result of Pearson’s correlation or Spearman's rank correlation for collinearity test. 19](#_Toc201567472)

[Supplementary Table 8: Log score for selecting best functional form. 20](#_Toc201567473)

[Appendix J: Population-adjusted prevalence 21](#_Toc201567474)

[Supplementary Table 9: Estimated yearly population-adjusted prevalence and number of people infected in Western Siberia and the Ural Region from 1975 to 2019, without prediction of western region due to sparse data. 21](#_Toc201567475)

[Appendix K: The temporal trend of prevalence in different sub-regions 23](#_Toc201567476)

[Supplementary Figure 2: Trends in the estimated prevalence of *O. felineus* infection in different sub-regions of Western Siberia and the Ural Region, without prediction of western region due to sparse data. BCI: Bayesian credible interval. 23](#_Toc201567477)

[Appendix L: Sensitivity analysis 24](#_Toc201567478)

[Supplementary File 7: Validation and comparison of joint model and prevalence-based model. 24](#_Toc201567479)

[Supplementary Table 10: Validation and comparison of joint model and prevalence-based model. 24](#_Toc201567480)

**Appendix A: PRISMA 2020**

### Supplementary File 1: PRISMA 2020 for Checklist

| **Section and Topic** | **Item #** | **Checklist item** | **Location where item is reported** |
| --- | --- | --- | --- |
| **TITLE** | | |  |
| Title | 1 | Identify the report as a systematic review. | Not applicable |
| ABSTRACT | | |  |
| Abstract | 2 | See the PRISMA 2020 for Abstracts checklist. | Not applicable |
| INTRODUCTION | | |  |
| Rationale | 3 | Describe the rationale for the review in the context of existing knowledge. | Introduction (page 4-5) |
| Objectives | 4 | Provide an explicit statement of the objective(s) or question(s) the review addresses. | Introduction (page 5) |
| METHODS | | |  |
| Eligibility criteria | 5 | Specify the inclusion and exclusion criteria for the review and how studies were grouped for the syntheses. | Method (“Disease data”: page 6), Appendix C |
| Information sources | 6 | Specify all databases, registers, websites, organisations, reference lists and other sources searched or consulted to identify studies. Specify the date when each source was last searched or consulted. | Method (“Disease data”: page 6), Appendix C |
| Search strategy | 7 | Present the full search strategies for all databases, registers and websites, including any filters and limits used. | Method (“Disease data”: page 6), Appendix C |
| Selection process | 8 | Specify the methods used to decide whether a study met the inclusion criteria of the review, including how many reviewers screened each record and each report retrieved, whether they worked independently, and if applicable, details of automation tools used in the process. | Method (“Disease data”: page 6), Appendix C |
| Data collection process | 9 | Specify the methods used to collect data from reports, including how many reviewers collected data from each report, whether they worked independently, any processes for obtaining or confirming data from study investigators, and if applicable, details of automation tools used in the process. | Method (“Disease data”: page 6), Appendix C |
| Data items | 10a | List and define all outcomes for which data were sought. Specify whether all results that were compatible with each outcome domain in each study were sought (e.g. for all measures, time points, analyses), and if not, the methods used to decide which results to collect. | Method (“Disease data”: page 6), Appendix C |
|  | 10b | List and define all other variables for which data were sought (e.g. participant and intervention characteristics, funding sources). Describe any assumptions made about any missing or unclear information. | Method (“Environmental, socioeconomic, and demographic data”: page 7), Appendix F (Supplementary Table 4, Supplementary Table 5) |
| Study risk of bias assessment | 11 | Specify the methods used to assess risk of bias in the included studies, including details of the tool(s) used, how many reviewers assessed each study and whether they worked independently, and if applicable, details of automation tools used in the process. | Method (“Disease data”: page 6), Appendix C (Supplementary File 3) |
| Effect measures | 12 | Specify for each outcome the effect measure(s) (e.g. risk ratio, mean difference) used in the synthesis or presentation of results. | Method (“Disease data”: page 6) |
| Synthesis methods | 13a | Describe the processes used to decide which studies were eligible for each synthesis (e.g. tabulating the study intervention characteristics and comparing against the planned groups for each synthesis (item #5)). | Method (“Statistical analysis”: page 7-8), Appendix B |
|  | 13b | Describe any methods required to prepare the data for presentation or synthesis, such as handling of missing summary statistics, or data conversions. | Method (“Statistical analysis”: page 7-8), Appendix G |
|  | 13c | Describe any methods used to tabulate or visually display results of individual studies and syntheses. | Method (“Statistical analysis”: page 7-8) |
|  | 13d | Describe any methods used to synthesize results and provide a rationale for the choice(s). If meta-analysis was performed, describe the model(s), method(s) to identify the presence and extent of statistical heterogeneity, and software package(s) used. | Method (“Statistical analysis”: page 8-9), Appendix G |
|  | 13e | Describe any methods used to explore possible causes of heterogeneity among study results (e.g. subgroup analysis, meta-regression). | Method (“Statistical analysis”: page 8-9), Appendix G |
|  | 13f | Describe any sensitivity analyses conducted to assess robustness of the synthesized results. | Appendix L |
| Reporting bias assessment | 14 | Describe any methods used to assess risk of bias due to missing results in a synthesis (arising from reporting biases). | Method (“Statistical analysis”: page 8-9) |
| Certainty assessment | 15 | Describe any methods used to assess certainty (or confidence) in the body of evidence for an outcome. | Method (“Statistical analysis”: page 8-9), Figure 3, Figure 4, Appendix J |
| RESULTS | | |  |
| Study selection | 16a | Describe the results of the search and selection process, from the number of records identified in the search to the number of studies included in the review, ideally using a flow diagram. | Results (“Data summaries”: page 10), Figure 1 |
|  | 16b | Cite studies that might appear to meet the inclusion criteria, but which were excluded, and explain why they were excluded. | Results (“Data summaries”: page 10), Figure 1 |
| Study characteristics | 17 | Cite each included study and present its characteristics. | Results (“Data summaries”: page 10), Table 1, Appendix D |
| Risk of bias in studies | 18 | Present assessments of risk of bias for each included study. | Supplementary Table 2**,** Appendix D |
| Results of individual studies | 19 | For all outcomes, present, for each study: (a) summary statistics for each group (where appropriate) and (b) an effect estimate and its precision (e.g. confidence/credible interval), ideally using structured tables or plots. | Table1, Figure 4 |
| Results of syntheses | 20a | For each synthesis, briefly summarise the characteristics and risk of bias among contributing studies. | Supplementary Table 2**,** Appendix D |
|  | 20b | Present results of all statistical syntheses conducted. If meta-analysis was done, present for each the summary estimate and its precision (e.g. confidence/credible interval) and measures of statistical heterogeneity. If comparing groups, describe the direction of the effect. | Figure 3, Figure 4, Appendix J |
|  | 20c | Present results of all investigations of possible causes of heterogeneity among study results. | Appendix K |
|  | 20d | Present results of all sensitivity analyses conducted to assess the robustness of the synthesized results. | Appendix L |
| Reporting biases | 21 | Present assessments of risk of bias due to missing results (arising from reporting biases) for each synthesis assessed. | Appendix K |
| Certainty of evidence | 22 | Present assessments of certainty (or confidence) in the body of evidence for each outcome assessed. | Appendix J |
| DISCUSSION | | |  |
| Discussion | 23a | Provide a general interpretation of the results in the context of other evidence. | Discussion (page 12) |
|  | 23b | Discuss any limitations of the evidence included in the review. | Discussion (page 12-13) |
|  | 23c | Discuss any limitations of the review processes used. | Discussion (page 12-13) |
|  | 23d | Discuss implications of the results for practice, policy, and future research. | Conclusion |
| OTHER INFORMATION | | |  |
| Registration and protocol | 24a | Provide registration information for the review, including register name and registration number, or state that the review was not registered. | Method (“Disease data”: page 6) |
|  | 24b | Indicate where the review protocol can be accessed, or state that a protocol was not prepared. | Appendix C |
|  | 24c | Describe and explain any amendments to information provided at registration or in the protocol. | Appendix C |
| Support | 25 | Describe sources of financial or non-financial support for the review, and the role of the funders or sponsors in the review. | Funding (page 17) |
| Competing interests | 26 | Declare any competing interests of review authors. | Conflict of Interest (page 17) |
| Availability of data, code and other materials | 27 | Report which of the following are publicly available and where they can be found: template data collection forms; data extracted from included studies; data used for all analyses; analytic code; any other materials used in the review. | Appendix C, Appendix E, Appendix F |

*From:*  Page MJ, McKenzie JE, Bossuyt PM, Boutron I, Hoffmann TC, Mulrow CD, et al. The PRISMA 2020 statement: an updated guideline for reporting systematic reviews. BMJ 2021;372:n71. doi: 10.1136/bmj.n71

For more information, visit: <http://www.prisma-statement.org/>

**Appendix B: Western Siberia and the Ural Region**

### **Supplementary File 2: Presentation of study area**

The study area in this paper, "Western Siberia and the Ural Region", is not an official composite region but rather a shorthand term for several administrative areas, which referred to other relevant literature [1].

### Supplementary Table 1: Administrative areas included in Western Siberia and the Ural Region.

| **Administrative area** | **Sub-region** |
| --- | --- |
| Khanty-Mansi Autonomous Okrug | Central |
| Tyumen Oblast | Central |
| Omsk Oblast | Central |
| Yamalo-Nenets Autonomous Okrug | Northen |
| Tomsk Oblast | Eastern |
| Kemerovo Oblast | Eastern |
| Novosibirsk Oblast | Eastern |
| Republic of Altai | Eastern |
| Altai Krai | Eastern |
| Chelyabinsk Oblast | Western |
| Kirov Oblast | Western |
| Kurgan Oblast | Western |
| Republic of Bashkortostan | Western |
| Republic of Tatarstan | Western |
| Perm Krai | Western |
| Republic of Udmurtia | Western |
| Sverdlovsk Oblast | Western |

**Appendix C: Study protocol**

### Supplementary File 3: Study protocol

| **Literature and epidemiological surveillance documents search**  **For survey data:**  **-**Peer-review via following databases.  - PubMed: search terms were “(Liver fluke* OR Opisthorchi*) AND (Russia* OR RF OR Siberia OR Ural)”.  - Web of Science: search terms were “(Liver fluke* OR Opisthorchi*) AND (Russia* OR RF OR Siberia OR Ural)”.  - eLIBRARY: search term was “Описторхоз*”.  - Scientific Medical Library of the Siberian State Medical University: search term was “Описторхоз*”.  -Google Scholar: general check for large disease database and other papers not included in the search engines.  - The literatures obtained from different sources was pooled in one reference database using EndNote X9.2 (Thomson Research Soft Ltd.) and duplicated ones were removed.  **For surveillance data:**  **-**On the official Russian website ‘ФЕДЕРАЛЬНАЯ СЛУЖБА ПО НАДЗОРУ В СФЕРЕ ЗАЩИТЫ ПРАВ ПОТРЕБИТЕЛЕЙ И БЛАГОПОЛУЧИЯ ЧЕЛОВЕКА’ with the URL: [rospotrebnadzor.ru](https://rospotrebnadzor.ru/), the incidence rate of *O. felineus* was collected from 17 ADM1 regions, respectively. | |
| --- | --- |
|  |  |
| **Exclusion criteria**  **For survey data:**  (a) in-vitro investigations, or absence of human studies; (b) with specific study designs (e.g., case-control studies, intervention studies without prevalence at baseline or control group) or specific population groups (e.g., patients, migrant population) that could not represent the status of infection risk in corresponding study locations; (c) with survey locations where preventive chemotherapy treatment took place within one year; (d) with study locations/areas not clearly identified, or conducted in islands far away from the mainland;  **For surveillance data:**  (a) the data includes neither the number of cases nor the incidence rate. | |
|  |  |
| **Inclusion criteria**  **For survey data:**  prevalence related community-based surveys (i.e., with information on number of examined and number of positive, or information on prevalence) conducted from 1975 onwards, data at provincial level and below, such as administrative divisions of level one (ADM1: Oblast, etc.), level two (ADM2: city, etc.), and point-level (village, etc.).  **For surveillance data:**  incidence data includes at least the incidence rate or the number of cases. | |
|  |  |
| **Identification of potentially relevant publications according to inclusion and exclusion criteria**  **For survey data:**  -Duplicates publications were checked and removed.  -Titles and abstracts were screened to identify potentially relevant articles.  -Full-text review to identify potentially relevant articles.  -During full text review, the potential relevant cited references of the articles were also screened to supplement the papers not collected earlier. | |
|  |  |
| **Quality control**  **For survey data:**  Quality control was undertaken by re-checking 20% of randomly selected irrelevant papers.  **For surveillance data:**  Quality control was undertaken by two individuals inputting data into Excel independently, followed by cross-checking. | |
|  |  |
| **Geolocation**  The coordinates of the survey sites were obtained from the corresponding publications or from Google Maps (<https://www.google.com/maps/>) if they were not provided by the corresponding publications. | |
|  |  |
| **Data extraction**  **For survey data:**  -Detailed information of the selected literatures were extracted, including literature information (e.g., journal, authors, publication date, title, volume and issue), survey information (e.g., survey type, survey time), location information (e.g., location names, location types, coordinates), and *O. felineus* infection data (e.g., diagnostic method, population type, the number of examined and positive, and percentage of positive).  -All extracted data were double-checked again to remove duplicates.  **-**For multiple papers with the same survey data, the data was extracted only once.  -If the study reported the corresponding prevalence of multiple locations in one province/city, all data were considered as separated point data. If multiple representative locations were sampled in one province/city, but the study only reported aggregated prevalence, this data was treated as areal data of the province/city.  **For surveillance data:**  **-**Detailed information from the epidemiological surveillance documents was extracted, including surveillance location, surveillance time, incidence rate, reported case numbers, and population demographics. | |
|  |  |
| **Contact authors**  Authors of publications were contacted in case where important information was missing (e.g., prevalence, number of examined, number of positive, diagnostic method, and survey year). | |
|  |  |
| **Quality assessment**  The quality assessment of each literature was undertaken using a nine-point checklist. The items of quality evaluation are as follows:  Q1: provide specific inclusion and exclusion criteria.  Q2: provide basic characteristics of the investigated population (gender, age, etc.).  Q3: provide prevalence rate of the survey.  Q4: provide number of positive patients and number of examined people of the survey.  Q5: provide diagnostic method used in the survey.  Q6: provide survey type.  Q7: provide time of the survey.  Q8: describe or discuss the possible bias of the survey or how confounders are controlled.  Q9: the literature comes from Science Citation Index Expanded database or Russia Citation Index.  Each item is scored 1 in case the publication meets) or 0 in contrary. The scores are summed up for all items and assigned to the publication as its quality score. | |
|  |  |
| **Data process and analysis**  **For survey data:**  -Data download and processing for environmental, socioeconomic data.  -Missing data imputation.  If survey year was missing, we assigned the year of publication minus one, which was the median of the duration from surveys conducted to results published in the collected survey data.  For surveys reported prevalence in intervals without exact observed values, the midpoints of the intervals were assigned.  **For surveillance data:**  -Missing data imputation.  If reported case numbers were missing, we multiplied the incidence rate for that year by demographic data as a substitute.  **Geostatistical analysis.**  An advanced multivariate Bayesian geostatistical modeling approach was developed to joint survey and surveillance data, covering both point- and areal-level information, incorporating potential influencing factors, spatial-temporal effects, and exchangeable non-spatial random effect. To be noted, prevalence data conducted in different years were not aggregated, rather be included in the modeling analysis as separated data. | |

**Appendix D: Quality assessment of eligible studies**

### Supplementary File 4: Quality assessment of eligible studies using an adapted nine-point quality assessment checklist

We did quality evaluation for each literature included in the final geostatistical modeling analysis, which was undertaken using an adapted nine-point quality assessment checklist.^1^ The items of quality evaluation are as follows:

Q1: provide specific inclusion and exclusion criteria.

Q2: provide basic characteristics of the investigated population (gender, age, etc.).

Q3: provide prevalence rate of the survey.

Q4: provide number of positive patients and number of examined people of the survey.

Q5: provide diagnostic method used in the survey.

Q6: provide survey type.

Q7: provide time of the survey.

Q8: describe or discuss the possible bias of the survey or how confounders are controlled.

Q9: the literature comes from Science Citation Index Expanded database or Russia Citation Index.

Each item is scored 1 in case the publication meets) or 0 in contrary. The scores are summed up for all items and assigned to the publication as its quality score.

### Supplementary Figure 1: The quality assessment of eligible studies.

Each piece represents the number and proportion of studies under the corresponding quality score.

### Supplementary Table 2: Result of quality assessment for eligible literatures.

| No. | Year | Author | Q1 | Q2 | Q3 | Q4 | Q5 | Q6 | Q7 | Q8 | Q9 | Score |
| --- | --- | --- | --- | --- | --- | --- | --- | --- | --- | --- | --- | --- |
| 1 | 2021 | Andrey Kozlova | 1 | 1 | 1 | 1 | 1 | 1 | 1 | 1 | 1 | 9 |
| 2 | 2021 | Fedorova, O. S. | 1 | 1 | 1 | 1 | 1 | 1 | 1 | 1 | 1 | 9 |
| 3 | 1980 | Beér, S.A. | 0 | 0 | 1 | 1 | 1 | 1 | 1 | 1 | 1 | 7 |
| 4 | 1992 | Kotelnikov, G. | 1 | 0 | 1 | 1 | 1 | 1 | 0 | 0 | 1 | 6 |
| 5 | 1986 | Ermolova, R. | 0 | 0 | 1 | 1 | 1 | 1 | 0 | 1 | 1 | 6 |
| 6 | 2011 | Ross | 1 | 0 | 1 | 1 | 1 | 1 | 1 | 1 | 1 | 8 |
| 7 | 1986 | Yahod, D | 0 | 0 | 1 | 1 | 1 | 1 | 1 | 0 | 1 | 6 |
| 8 | 1982 | Merzlova, N.B. | 0 | 0 | 1 | 1 | 1 | 1 | 0 | 0 | 1 | 5 |
| 9 | 1988 | Uchuatkin, E. | 0 | 0 | 1 | 1 | 1 | 1 | 1 | 1 | 1 | 7 |
| 10 | 1985 | Bronshtein, A.M. | 0 | 0 | 1 | 1 | 1 | 1 | 1 | 1 | 1 | 7 |
| 11 | 2013 | Bobyreva, N. | 1 | 0 | 1 | 1 | 1 | 1 | 1 | 0 | 1 | 7 |
| 12 | 1983 | Borodina, O.N. | 0 | 0 | 1 | 1 | 1 | 1 | 1 | 0 | 1 | 6 |
| 13 | 2005 | Il’inskih, E. | 1 | 0 | 1 | 1 | 1 | 1 | 0 | 0 | 1 | 6 |
| 14 | 1989 | Zhuravlev, S.E. | 0 | 0 | 1 | 1 | 1 | 1 | 1 | 0 | 1 | 6 |
| 15 | 2004 | Tkachenko, T. | 1 | 0 | 1 | 1 | 1 | 1 | 1 | 1 | 1 | 8 |
| 16 | 1993 | Mefod’ev, V.V. | 1 | 0 | 1 | 1 | 1 | 1 | 1 | 1 | 1 | 8 |
| 17 | 1991 | Bronshtein, A.M. | 1 | 0 | 1 | 1 | 1 | 1 | 0 | 0 | 1 | 6 |
| 18 | 1985 | Bronshtein, A.M. | 0 | 0 | 1 | 1 | 1 | 1 | 1 | 0 | 1 | 6 |
| 19 | 1986 | Bronshtein, A.M. | 0 | 0 | 1 | 1 | 1 | 1 | 1 | 0 | 1 | 6 |
| 20 | 1986 | Bronshtein, A.M. | 0 | 0 | 1 | 1 | 1 | 1 | 0 | 1 | 1 | 6 |
| 21 | 1987 | Bronshtein, A.M. | 1 | 0 | 1 | 1 | 1 | 1 | 1 | 0 | 1 | 7 |
| 22 | 2010 | Starostina, O.j. | 1 | 0 | 1 | 1 | 1 | 1 | 1 | 0 | 1 | 7 |
| 23 | 1998 | Kotelkin, A. | 1 | 0 | 1 | 1 | 1 | 1 | 0 | 0 | 1 | 6 |
| 24 | 1979 | Zavokin, V.D. | 0 | 0 | 1 | 1 | 1 | 1 | 1 | 0 | 1 | 6 |
| 25 | 1990 | Balasheva, I.I. | 1 | 0 | 1 | 0 | 1 | 1 | 1 | 0 | 1 | 6 |
| 26 | 1995 | Bychkova, N. | 1 | 0 | 1 | 1 | 1 | 1 | 0 | 0 | 1 | 6 |
| 27 | 2000 | Lepehin, A. | 1 | 0 | 1 | 1 | 1 | 1 | 0 | 0 | 1 | 6 |
| 28 | 1998 | Mikhailov, M. | 1 | 0 | 1 | 1 | 1 | 1 | 0 | 1 | 1 | 7 |
| 29 | 2007 | Ogorodova, L.M. | 1 | 1 | 1 | 1 | 1 | 1 | 0 | 1 | 1 | 8 |
| 30 | 2006 | Il’inskih | 1 | 0 | 1 | 1 | 1 | 1 | 0 | 0 | 1 | 6 |
| 31 | 2011 | Ogorodova, L. | 1 | 1 | 1 | 1 | 1 | 1 | 1 | 1 | 1 | 9 |
| 32 | 1984 | Klebanovski, V.A. | 0 | 0 | 1 | 1 | 1 | 1 | 0 | 1 | 1 | 6 |
| 33 | 1990 | Klebanovskaja | 0 | 0 | 1 | 1 | 1 | 1 | 1 | 0 | 1 | 6 |
| 34 | 1999 | Musyrgalina, F. | 1 | 0 | 1 | 1 | 1 | 1 | 0 | 0 | 1 | 6 |
| 35 | 1994 | T N Tsybina | 0 | 0 | 1 | 1 | 1 | 1 | 1 | 0 | 1 | 6 |
| 36 | 1989 | V D Zavoìkin | 0 | 0 | 1 | 1 | 1 | 1 | 0 | 1 | 1 | 6 |
| 37 | 2020 | Proskurina, L | 1 | 0 | 1 | 1 | 1 | 1 | 1 | 1 | 1 | 8 |

**Appendix E: Surveillance data sources**

### Supplementary Table 3: Sources and access dates of surveillance data.

| Geographical units | Sources | Access dates |
| --- | --- | --- |
| Altai Krai | [http://22.rospotrebnadzor.ru](http://22.rospotrebnadzor.ru/) | 2024.09 |
| Chelyabinsk Oblast | <http://74.rospotrebnadzor.ru> | 2024.09 |
| Kemerovo Oblast | <http://42.rospotrebnadzor.ru> | 2024.09 |
| Khanty-Mansi Autonomous Okrug | <http://86.rospotrebnadzor.ru> | 2024.09 |
| Kirov Oblast | <http://43.rospotrebnadzor.ru> | 2024.09 |
| Kurgan Oblast | <http://45.rospotrebnadzor.ru> | 2024.09 |
| Novosibirsk Oblast | <http://54.rospotrebnadzor.ru> | 2024.09 |
| Omsk Oblast | <http://55.rospotrebnadzor.ru> | 2024.09 |
| Perm Krai | <http://59.rospotrebnadzor.ru> | 2024.09 |
| Republic of Altai | <http://04.rospotrebnadzor.ru> | 2024.09 |
| Republic of Bashkortostan | <http://02.rospotrebnadzor.ru> | 2024.09 |
| Republic of Tatarstan | <http://16.rospotrebnadzor.ru> | 2024.09 |
| Republic of Udmurtia | <http://18.rospotrebnadzor.ru> | 2024.09 |
| Sverdlovsk Oblast | <http://66.rospotrebnadzor.ru> | 2024.09 |
| Tomsk Oblast | <http://70.rospotrebnadzor.ru> | 2024.09 |
| Tyumen Oblast | <http://72.rospotrebnadzor.ru> | 2024.09 |
| Yamalo-Nenets Autonomous Okrug | <http://89.rospotrebnadzor.ru> | 2024.09 |

**Appendix F: Covariate**

### Supplementary Table 4: Remote sensing data sources^a^

| Source | Data type | Data period | Temporal resolution | Spatial resolution |
| --- | --- | --- | --- | --- |
| MODIS/Terra^b^ | LST^c^ | 2000-2019 | 8 days | 1km |
| MODIS/Terra^b^ | NDVI^d^ | 2000-2019 | 16 days | 1km |
| MODIS/Terra^b^ | Land cover | 2001-2019 | Yearly | 1km |
| DIVA-GIS^e^ | Elevation | 2000 | - | 1km |
| WorldClim^f^ | Annual precipitation | 1980-2019 | - | 1km |
| DIVA-GIS^e^ | Distance to the nearest open water bodies | 2000 | - | 30m |
| The Atlas of the Biosphere^g^ | Soil moisture | 1950-1999 | - | 50km |
| Worldpop^h^ | Population | 2000-2019 | - | 1km |
| SEDAC^i^ | HII^j^ | 1995-2004 | - | 1km |
| Malaria atlas^k^ | Travel time to the nearest big city | 2015 | - | 1km |
| Russian Federal State Statistics Service^l^ | Population growth rate | - | - | ADM1-level |
| ^a^Data asccessed in March 2022.  ^b^Moderate Resolution Imageing Spectroradiometer (MODIS) /Terra, available at: <https://lpdaac.usgs.gov/>.  ^c^Land surface temperature (LST) in the daytime and at night.  ^d^NDVI: Normalized difference vegetation index.  ^e^Available at: https://www.diva-gis.org/gdata/.  ^f^Available at: <http://www.worldclim.org/current>/.  ^g^Available at: <http://www.sage.wisc.edu/atlas/>.  ^h^Available at: <https://www.worldpop.org/geodata/>.  ^i^Socioeconomic Data and Applications Center, available at: <http://sedac.ciesin.org/>.  ^j^HII: Human influence index.  ^k^D.J. Weiss, A. Nelson, et al. A global map of travel time to cities to assess inequalities in accessibility in 2015. Nature (2018). doi:10.1038/nature25181, available at: [https://map.ox.ac.uk/res earch-project/accessibility_to_cities/](https://map.ox.ac.uk/res%20earch-project/accessibility_to_cities/)  ^l^Available at: https://showdata.gks.ru/report/278930/. | | | | |

### **Supplementary Table 5: The spatial covariates used in the present stud**y

| Variable | Overall | Central | Eastern | Northen |
| --- | --- | --- | --- | --- |
| LST | -1.3 (-1.2, 2.4) | -0.1 (-2.0, 2.4) | 2.7 (0.8, 5.3) | -6.3 (-8.5, -4.6) |
| NDVI | 0.40 (0.29, 0.47) | 0.45 (0.39, 0.50) | 0.45 (0.40, 0.52) | 0.27 (0.21, 0.33) |
| Elevation | 85 (46, 126) | 85 (58, 109) | 139 (109, 240) | 41 (21, 67) |
| Annual precipitation | 509 (427, 545) | 532 (498, 551) | 526 (456, 563) | 449 (325, 512) |
| Soil moisture | 65.4 (46.1, 74.7) | 73.2 (60.6, 77.6) | 55.2 (38.4, 66.8) | 67.6 (42.0, 73.8) |
| Population | 0.22 (0.05, 1.47) | 0.38 (0.11, 1.92) | 1.08 (0.25, 3.53) | 0.04 (0.02, 0.13) |
| HII^j^ | 4.0 (0.0, 8.9) | 4.0 (0.0, 8.4) | 9.5 (4.0, 16.3) | 1.6 (0.0, 4.0) |
| Travel time to the nearest big city | 630 (281, 1223) | 442 (217, 798) | 382 (199, 815) | 1230 (702, 2004) |

**Appendix G:** **Model fitting**

### **Supplementary File 5: Model fitting**

**Reducing the computational burden by defining the time knots**

The survey period of this study was from 1975 to 2019. We built the Gaussian Markov random field (GMRF) on regular temporal knots under the SPDE framework to decrease the computational burden, that is, $\omega=(\omega_{t}=1975,\omega_{t}=1986,\omega_{t}=1997, \omega_{t}=2008, \omega_{t}=2019)$. The latent fields in other years are approximated by employing the B-spline basis function of degree two for the projection of $\omega$, that is $B_{i,1}\left( t \right)=\left\{ \begin{aligned} 1, t_{i}\leq t<t_{i+1} \\ 0, otherwise \end{aligned} \right.$ and$B_{i,m}\left( t \right)=\frac{t-t_{i}}{t_{i+m-1}-t_{i}}B_{i,m-1}\left( t \right)+\frac{t_{i+m}-t}{t_{i+m}-t_{i+1}}B_{i+1,m-1}\left( t \right)$, where the degree $m$ is equal to 2.[2, 3]

**Prior distributions**

Based on previous literature, the models were built under a Bayesian framework. Minimally informative priors were specified for regression coefficients, two precision parameters, temporal correlation coefficient, range parameter, tuning coefficient as follows: $\beta\sim N\left( 0, {10}^{5}\boldsymbol{I} \right),$ , $\log\left( 1/{\sigma_{\phi}^{2}} \right)\sim\mathrm{logGamma}\left( 1,0.01 \right)$, $\log\left( 1/{\sigma_{nonsp}^{2}} \right)\sim\mathrm{logGamma}\left( 1,0.01 \right)$ $\log\left( \left( 1+\rho\right)/\left( 1-\rho\right) \right)\sim N\left( 0,0.15 \right)$, $\log\left( \kappa\right)\sim N({\log\left( \sqrt{8} \right)}/{d,1)}$ and $\alpha\sim N\left( 0,2 \right)$ where$d$is the median distance between the predicted grids.

**Appendix H: Surveillance data**

### **Supplementary Table 6: Overview of *O. felineus* infection surveillance data in Western Siberia and the Ural Region of Russian Federation.**

| Geographical units | Available time period | Absolute number | Annual mean incidence  (per 100,000) |
| --- | --- | --- | --- |
| Altai Krai | 1997-2019 | 33,075 | 53.79 |
| Chelyabinsk Oblast | 2001-2019 | 6,077 | 10.32 |
| Kemerovo Oblast | 2003-2019 | 29,049 | 63.53 |
| Khanty-Mansi Autonomous Okrug | 2002-2019 | 160,351 | 558.81 |
| Kirov Oblast | 2000-2019 | 1,529 | 3.76 |
| Kurgan Oblast | 1999-2019 | 12,915 | 63.89 |
| Novosibirsk Oblast | 1991-2019 | 94,991 | 121.53 |
| Omsk Oblast | 2007-2019 | 26,146 | 98.61 |
| Perm Krai | 2001-2019 | 3,516 | 6.41 |
| Republic of Altai | 2006-2019 | 3,091 | 52.36 |
| Republic of Bashkortostan | 1997-2006 2008-2019 | 497 | 0.55 |
| Republic of Tatarstan | 2011-2019 | 214 | 0.67 |
| Republic of Udmurtia | 2005-2019 | 463 | 1.95 |
| Sverdlovsk Oblast | 2010-2017  2019-2019 | 9,525 | 23.76 |
| Tomsk Oblast | 1999-2019 | 86,942 | 362.50 |
| Tyumen Oblast | 2000-2019 | 63,293 | 217.86 |
| Yamalo-Nenets Autonomous Okrug | 2004-2019 | 22,160 | 258.53 |
| Total | / | 553,834 | 82.88 |

**Appendix I: The variable selection**

### **Supplementary File 6: The** **collinearity, optimal functional form and selection of variable.**

Firstly, to prevent collinearity, we calculated Pearson’s or Spearman correlation coefficients for each pair of continuous variables. If the absolute values of correlation coefficients exceeded 0.8, we only retained the one more meaningful or with higher-quality in each pair [4]. Secondly, we selected the functional form of each continuous variable with the minimum Deviance Information Criterion (DIC) [5]. Finally, through the screening method of full subsets, the subset with the smallest DIC was finally selected: the travel time of the nearest big city (continuous type), the normalized vegetation index (binary variable), the distance to the nearest water body (binary variable), and the altitude (binary variable) [6-8].

### Supplementary Table 7: The result of Pearson’s correlation or Spearman's rank correlation for collinearity test.

| Variable | LST | NDVI | Elevation | Annual precipitation | Distance to the nearest open water bodies | Soil moisture | HII | Travel time to the nearest big city |
| --- | --- | --- | --- | --- | --- | --- | --- | --- |
| LST | 1 | 0.0410 | 0.4419 | -0.2968 | 0.1415 | -0.3819 | 0.4126 | -0.6124 |
| NDVI | 0.0410 | 1 | 0.3179 | -0.0749 | 0.2406 | -0.0266 | -0.3760 | 0.0424 |
| Elevation | 0.4419 | 0.3179 | 1 | -0.0618 | 0.3675 | -0.2328 | 0.0979 | -0.2491 |
| Annual precipitation | -0.2968 | -0.0750 | -0.0618 | 1 | -0.2377 | 0.5512 | 0.1151 | 0.0536 |
| Distance to the nearest open water bodies | 0.1415 | 0.2406 | 0.3675 | -0.2377 | 1 | -0.0206 | -0.3233 | -0.0380 |
| Soil moisture | -0.3819 | -0.0266 | -0.2328 | 0.5513 | -0.0206 | 1 | -0.1896 | -0.5210 |
| HII | 0.4126 | -0.3760 | 0.0979 | 0.1152 | -0.3233 | -0.1896 | 1 | 0.1496 |
| Travel time to the nearest big city | -0.6124 | 0.0424 | -0.2491 | 0.0536 | -0.0380 | -0.5206 | 0.1496 | 1 |

### Supplementary Table 8: Log score for selecting best functional form.

| Variable | Continuous variable | Binary variable | Three categorical variable |
| --- | --- | --- | --- |
| LST | 56.0963 | 56.1009 | 56.0990 |
| NDVI | 56.1026 | 56.1012 | 56.1027 |
| Elevation | 56.1111 | 56.1019 | 56.1052 |
| Annual precipitation | 56.1042 | 56.1008 | 56.1028 |
| Distance to the nearest open water bodies | 56.1027 | 56.1005 | 56.1055 |
| Soil moisture | 56.0873 | 56.1027 | 56.0934 |
| HII | 56.0959 | 56.1008 | 56.1019 |
| Travel time to the nearest big city | 56.0967 | 56.1009 | 56.1022 |

**Appendix J: Population-adjusted prevalence**

### Supplementary Table 9: Estimated yearly population-adjusted prevalence and number of people infected in Western Siberia and the Ural Region from 1975 to 2019, without prediction of western region due to sparse data.

| **Year of survey** | **Estimated prevalence**  **(%, 95% BCI)** | **Estimated number of people infected**  **(95% BCI)**$\boldsymbol{\times}\boldsymbol{10}^{\boldsymbol{6}}$ |
| --- | --- | --- |
| 1975 | 31.86 (20.50-49.60) | 5.38 (3.46-8.38) |
| 1976 | 31.85 (20.51-49.53) | 5.38 (3.46-8.36) |
| 1977 | 31.64 (20.44-49.25) | 5.34 (3.45-8.31) |
| 1978 | 31.38 (20.34-48.19) | 5.30 (3.43-8.13) |
| 1979 | 31.16 (20.04-47.74) | 5.26 (3.38-8.05) |
| 1980 | 30.75 (19.49-46.86) | 5.19 (3.28-7.90) |
| 1981 | 30.38 (19.03-46.09) | 5.12 (3.20-7.76) |
| 1982 | 29.75 (18.41-45.65) | 5.01 (3.10-7.69) |
| 1983 | 29.29 (17.45-45.54) | 4.93 (2.93-7.66) |
| 1984 | 28.37 (16.36-44.91) | 4.78 (2.75-7.56) |
| 1985 | 27.40 (15.35-45.45) | 4.61 (2.58-7.64) |
| 1986 | 27.07 (13.85-46.76) | 4.55 (2.33-7.86) |
| 1987 | 27.03 (12.53-48.28) | 4.54 (2.10-8.12) |
| 1988 | 26.82 (11.86-49.52) | 4.51 (1.99-8.32) |
| 1989 | 26.87 (11.60-50.86) | 4.51 (1.95-8.55) |
| 1990 | 26.99 (11.62-51.23) | 4.53 (1.95-8.61) |
| 1991 | 27.20 (11.60-51.48) | 4.57 (1.95-8.65) |
| 1992 | 27.62 (11.72-51.94) | 4.64 (1.97-8.72) |
| 1993 | 27.82 (12.19-51.77) | 4.67 (2.04-8.70) |
| 1994 | 28.07 (12.77-51.49) | 4.71 (2.14-8.65) |
| 1995 | 28.46 (13.75-49.51) | 4.78 (2.31-8.31) |
| 1996 | 29.17 (15.37-47.60) | 4.90 (2.58-7.99) |
| 1997 | 30.01 (17.25-46.97) | 5.04 (2.89-7.89) |
| 1998 | 31.07 (18.37-48.33) | 5.22 (3.08-8.11) |
| 1999 | 32.17 (19.09-50.18) | 5.40 (3.20-8.42) |
| 2000 | 33.29 (19.93-51.21) | 5.59 (3.34-8.60) |
| 2001 | 34.30 (21.55-52.65) | 5.75 (3.61-8.83) |
| 2002 | 35.23 (21.62-54.16) | 5.89 (3.61-9.06) |
| 2003 | 36.03 (22.16-54.30) | 6.01 (3.69-9.06) |
| 2004 | 36.39 (23.26-54.07) | 6.04 (3.86-8.98) |
| 2005 | 37.12 (23.26-54.30) | 6.13 (3.84-8.97) |
| 2006 | 37.72 (23.19-54.34) | 6.20 (3.81-8.94) |
| 2007 | 37.59 (21.98-56.78) | 6.17 (3.61-9.33) |
| **Year of survey** | **Estimated prevalence**  **(%, 95% BCI)** | **Estimated number of people infected**  **(95% BCI)**$\boldsymbol{\times}\boldsymbol{10}^{\boldsymbol{6}}$ |
| 2008 | 38.17 (19.90-59.00) | 6.28 (3.27-9.70) |
| 2009 | 38.50 (18.06-60.99) | 6.34 (2.97-10.05) |
| 2010 | 38.87 (16.30-63.86) | 6.42 (2.69-10.55) |
| 2011 | 39.48 (15.96-64.33) | 6.55 (2.64-10.67) |
| 2012 | 40.24 (16.24-66.19) | 6.71 (2.71-11.04) |
| 2013 | 40.85 (15.89-67.26) | 6.84 (2.66-11.27) |
| 2014 | 41.81 (16.36-69.19) | 7.03 (2.75-11.64) |
| 2015 | 42.84 (17.20-70.59) | 7.23 (2.90-11.91) |
| 2016 | 43.91 (17.81-71.67) | 7.43 (3.01-12.13) |
| 2017 | 44.41 (16.91-73.26) | 7.53 (2.87-12.43) |
| 2018 | 45.77 (15.58-74.29) | 7.76 (2.64-12.61) |
| 2019 | 46.61 (15.09-76.50) | 7.91 (2.56-12.98) |

**Appendix K: The temporal trend of prevalence in different sub-regions**

### Supplementary Figure 2: Trends in the estimated prevalence of *O. felineus* infection in different sub-regions of Western Siberia and the Ural Region, without prediction of western region due to sparse data. BCI: Bayesian credible interval. (a) Central part of study area, (b) Eastern part of study area, (c) Northen part of study area.

**Appendix L: Sensitivity analysis**

### Supplementary File 7: Validation and comparison of joint model and prevalence-based model.

To better substantiate the prediction of the joint model, we conducted a sensitivity analysis by comparing with a based prevalence model. To evaluate the performance of the models, we calculated mean absolute error (MAE), mean square error (MSE) and the area under the receiver-operating characteristic (ROC) curve (AUC). Instead of using leave-one-out cross-validation, we directly compared models fitted with the full dataset.

### Supplementary Table 10: Validation and comparison of joint model and prevalence-based model.

|  | **Joint model** | **Based model** |
| --- | --- | --- |
| MAE | 0.097 | 0.110 |
| MSE | 0.016 | 0.026 |
| AUC | 0.901 | 0.857 |

**Reference:**

1. Fedorova, O.S., et al., *Opisthorchis felineus infection prevalence in Western Siberia: A review of Russian literature.* Acta Trop, 2018. **178**: p. 196-204.

2. Cameletti, M., et al., *Spatio-temporal modeling of particulate matter concentration through the SPDE approach.* Adv Stat Anal, 2013. **97**(2): p. 109-131.

3. Krainski, E., et al., *Advanced Spatial Modeling with Stochastic Partial Differential Equations Using R and INLA*. 2019, Boca Raton: CRC Press, Taylor & Francis Group.

4. Dormann, C.F., et al., *Collinearity: a review of methods to deal with it and a simulation study evaluating their performance.* ECOGRAPHY, 2013. **36**(1): p. 27-46.

5. Pettit, L.J.J.o.t.R.S.S.S.B., *The conditional predictive ordinate for the normal distribution.* 1990. **52**(1): p. 175-184.

6. Brooker, S., et al., *Predicting the distribution of urinary schistosomiasis in Tanzania using satellite sensor data.* Trop Med Int Health, 2001. **6**(12): p. 998-1007.

7. Xiao, H.Y., et al., *The spatial-temporal risk profiling of Clonorchis sinensis infection over 50 years implies the effectiveness of control programs in South Korea: a geostatistical modeling study.* Lancet Reg Health West Pac, 2023. **33**: p. 100697.

8. Zhao, T.T., et al., *Model-based spatial-temporal mapping of opisthorchiasis in endemic countries of Southeast Asia.* Elife, 2021. **10**.
